# Dengue Disease Surveillance: Improving Data for Dengue Control

**DOI:** 10.1371/journal.pntd.0003311

**Published:** 2014-11-06

**Authors:** Olaf Horstick, Amy C. Morrison

**Affiliations:** 1 Institute of Public Health, University of Heidelberg, Heidelberg, Germany; 2 Department of Entomology, University of California, Davis, Davis, California, United States of America; 3 Naval Medical Research Unit No. 6, Washington, D.C., United States of America; Duke-NUS, Singapore

Dengue disease surveillance has been notoriously difficult in the past, with differences between reported and estimated cases being very large; since the quality of available data is generally poor, case estimates vary widely [1], [2]. While it is clear that the incidence of dengue is on the increase worldwide, it is difficult to determine how much of this increase should be attributed to improvements in surveillance systems versus increased transmission and disease burden. Further complicating the interpretation of surveillance data are (1) differences in laboratory confirmation rates, (2) dynamics in treatment-seeking behaviour, and (3) changes in case definitions and classifications. Laboratory confirmation of cases is often limited [3] because of cost and the requirement for technical expertise. Although there are an increasing number of rapid diagnostic tests available, their sensitivity and specificity can vary dramatically from those reported by the manufacturer and across endemic settings [4], [5]. There is a need for diagnostic tests that are cheaper and easier to use than those that are currently available. However, even given accurate diagnostics, studies of health-seeking behaviour dynamics are urgently needed to interpret case counts. One factor influencing health-seeking behaviour is disease severity; while the majority of dengue infections are thought to be asymptomatic [2], they are nonetheless important because of their contribution to transmission. Case definitions and case classifications for dengue have varied [6], complicating the interpretation of surveillance data collected before and after the change; although the new case definitions and classifications [1] may have improved patient management, their impact on the study of the biology of dengue disease remains controversial. In summary, only few dengue cases are diagnosed and even fewer are confirmed with a diagnostic test—for these reasons the true burden of disease must rely on estimates rather than counts of reported dengue cases.

Anticipation of the licensure of a dengue vaccine has brought new urgency to the need to establish and improve dengue disease surveillance programmes—these needs were described by the World Health Organization's Scientific Working Group in 2005 (WHO/TDR) [7]. Clinical Phase IV vaccine studies will depend on these systems to evaluate the effectiveness of different products when applied on a large scale and, more importantly, to evaluate lingering concerns about the potential contribution of a vaccine to the risk of severe disease [8]. Without understanding the epidemiological context in which vaccines are deployed, accessing efficacy is severely limited because exposure to dengue virus, which is known to be extremely heterogeneous, is unknown. Entomological surveillance can provide a partial estimate of exposure or risk, and future dengue prevention programmes will require an integrated approach combining vaccine deployment, vector control, good clinical management, therapeutics, and community involvement. The Partnership for Dengue Control (PDC), a new public health initiative hosted by the Fondation Mérieux, grew out of the Sanofi Pasteur v2V programme to facilitate the introduction of dengue vaccines into endemic countries, with a new mission to “promote development and implementation of innovative, integrated, synergistic approaches for the prevention and control of dengue” (http://www.fondation-merieux.org/fondation-merieux-hosts-the-pdc-a-novel-partnership-for-dengue-control). A cornerstone of this new initiative is surveillance.

This issue of *PLOS Neglected Tropical Diseases* includes a collection of five systematic reviews from the French-speaking Caribbean, Malaysia, Mexico, Thailand, and the Philippines, analysing all available epidemiological studies from 2000–2012 and following a common protocol and a previously published pilot study from Brazil. These studies have been sponsored by the pharmaceutical company Sanofi, which is heavily involved in dengue vaccine development. Their aims are to improve dengue surveillance, both for estimating a better baseline for a possible vaccine rollout and to improve the quality of clinical studies. Further country studies with a similar design are currently under way.

The studies published here follow the methodology of systematic reviews as outlined in the PRISMA statement [9]. The advantages of a systematic review include the exhaustive description of existing answers of a given research question, the inclusion of studies that are not directly comparable, the ability to provide authoritative public health recommendations, and the opportunity to identify knowledge gaps. The presented studies from these five countries and the studies that are forthcoming are especially strong in the descriptive part of the analysis, perhaps less so in the analytical components. When including the pilot study from Brazil, the systematic reviews show a common pattern of shortcomings of surveillance systems that warrant examination. These common patterns and conclusions are summarised in the following Expert Commentary from a group of the main authors.

Without a good estimate of burden of disease, it is difficult to raise awareness and funds for research [10]. New approaches and metrics for quantifying the relationship between data captured from passively obtained case counts and the actual number of people contributing to transmission are needed. Efforts to develop correction factors [11], as well as more sophisticated modelling approaches [12]–[15], have been developed but require validation. This is only possible if surveillance data are related to more rigorously collected information from active surveillance studies or longitudinal cohort studies in which all infections are counted, including laboratory-confirmed asymptomatic and non-severe cases. Beyond the burden of disease, models of vaccine deployment and vector control or combinations of both will require estimates of all infections extrapolated from local surveillance data for parameterisation. The relationship between detected cases and inapparent infections is complicated and represents a major challenge to improving dengue surveillance.

Although the limitations and variation in dengue surveillance systems are clear, the lack of coordination between surveillance and response is demonstrated by the observation that even with the information available, dengue control is often not implemented [16], routine control methods are often not applied [17], and even when implemented, control measures are often not executed comprehensively or properly. Methods for outbreak prediction and detection vary across countries and lack standardisation [18], but as a minimum, standard dengue control should be rolled out in outbreaks, both to reorganise health services for a potentially massive increase of dengue cases and to implement emergency vector control—good disease surveillance is a prerequisite. Previous comparative studies of surveillance systems and guidelines application [19] have shown that “(1) inaccessibility of dengue guidelines, (2) lack of training, (3) insufficient number of staff to correctly apply the guidelines at the frontline, and (4) the unavailability of diagnostic tests” are all issues that need addressing.

Improving surveillance along the lines suggested by the authors of the presented systematic reviews is clearly not sufficient if the response to dengue control is not also adequately strengthened. Enhanced dengue control must identify and validate optimal combinations of vector control, clinical management, and in the future, dengue vaccines and drugs that provide the highest probability of success in a given ecological and epidemiological context.
